# A case report of an alcoholic with delirium tremens diagnosed with hypopharyngeal cancer: Psychiatrists played important roles in the smooth diagnosis and treatments

**DOI:** 10.1002/pcn5.54

**Published:** 2022-11-09

**Authors:** Tetsuro Ishida, Tomonori Murayama, Seiju Kobayashi

**Affiliations:** ^1^ Department of Psychiatry Japan Health Care College Sapporo Japan; ^2^ Department of Psychiatry Asahikawa Keisenkai Hospital Asahikawa Japan; ^3^ Department of Psychiatry Shinyukai Nakae Hospital Sapporo Japan

**Keywords:** alcoholism, case report, delirium tremens, hypopharyngeal cancer, shared decision‐making

## Abstract

**Background:**

Alcohol use increases the risk of developing several types of cancer. Of these, hypopharyngeal cancer has one of the worst prognoses. Moreover, treating an alcoholic patient with hypopharyngeal cancer is often difficult. There are various treatments for hypopharyngeal cancer, including surgery, chemotherapy, and radiotherapy, depending on the state of the cancer and the patient's quality of life. Patients need physical, psychological, and social support in decision‐making and post‐treatment follow‐up. This is especially true for alcoholic patients.

**Case Presentation:**

A 59‐year‐old man was admitted to our hospital with complaints of fatigue, loss of appetite, and tremor of the upper limbs. He was single, alcoholic, and had no family. After treatment for delirium tremens, he complained of throat pain. After endoscopy, magnetic resonance imaging, and examination with 18F‐fluorodeoxyglucose positron emission tomography/computed tomography, he was diagnosed with Stage 4 A (T2 N2c M0) hypopharyngeal cancer. The psychiatrist and otolaryngologist discussed the patient's decision‐making capacity and the various risks associated with treatment. Shared decision‐making with the patient was considered most important in determining the treatment strategy. As a result, the patient decided to receive endoscopic laryngopharyngeal surgery in combination with lymphadenectomy, a challenging surgical treatment. The operation was successful, and the patient is now ready for a new life after discharge.

**Conclusion:**

Psychiatrists have a significant role to play in the oncological treatment of patients with alcoholism and other psychiatric disorders.

## Graphical abstract

A 59‐year‐old alcoholic man with delirium tremens was diagnosed as having hypopharyngeal cancer. Liaison and shared decision‐making between the patient, psychiatrist, and otolaryngologist ensured smooth diagnosis and treatments with consideration for the patient's quality of life.

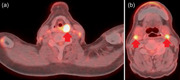

## BACKGROUND

Alcohol use increases the risk of developing several types of cancer. The most common examples are cancers of the breast, colon, rectum, stomach, and liver, as well as head and neck cancer.^1^ Hypopharyngeal cancer has one of the worst prognoses of all head and neck cancers. The 5‐year overall survival rate for patients is approximately 30%–35%.^2^ Therefore, hypopharyngeal cancer requires rapid diagnosis and appropriate treatment. In clinical practice, however, it is often difficult for otolaryngologists to treat hypopharyngeal cancer patients with alcohol use disorder. This is also true for alcoholics with other diseases requiring surgery, but alcoholics with hypopharyngeal cancer are at higher risk of perioperative delirium,^3^ and alcoholic hepatitis interferes with chemotherapy. We present a case of an alcoholic patient who presented with delirium tremens. After the delirium tremens resolved, the patient was diagnosed with hypopharyngeal cancer and underwent successful surgery. We hope that this report will be helpful to patients in similar circumstances and to the psychiatrists who treat them.

## CASE PRESENTATION

A 59‐year‐old man was admitted to our hospital with complaints of fatigue, loss of appetite, and tremor of the upper limbs. After being diagnosed with alcoholism at our hospital 15 years prior, and irregular visits for treatment, he had had periods of intermittent sobriety, but these had stopped, and he had been drinking approximately 120 g/day of alcohol (mainly shochu) every day for 3 years. The patient's last drink was 2 days before admission to the hospital. The day before admission, he was unable to drink because of malaise. He had obesity, hyperlipidemia, and liver dysfunction. He had previously been a fisherman and had lived with his wife. He had been laid off because of his alcoholism and his family life deteriorated, leading to divorce. He then lived on welfare. His parents had previously died, and none of his three older brothers could be contacted. He had no dependents.

Upon admission, the patient was 174 cm tall and weighed 74 kg (down from 80 kg 1 year earlier). His skin was damp with cold sweat. His temperature was 35.3°C, blood pressure was 157/108 mmHg, pulse rate was 93 beats/min, and saturation of percutaneous oxygen was 96% (room air). His conscious state was assessed as E4/4, V4/5, M6/6 on the Glasgow Coma Scale.^4^ He was able to greet people and briefly respond, but showed disorientation. He did not show excitement or agitation but claimed to be tormented by anxiety and nightmares. He had no hallucinations or agitation. His bilateral upper limbs showed fine tremor. There were no abnormal neurological findings, such as ataxia or abnormal tendon reflexes.

On the basis of the above elements, he was diagnosed with mild alcohol withdrawal delirium associated with alcohol withdrawal. From the day of admission (Day 1), he was given intravenous fluids, including glutathione, flavin adenine dinucleotide sodium and vitamin B complex for 1 week. As a result, his consciousness improved, and he no longer had tremors in the upper limbs.

However, on Day 16, he complained of a sore throat when swallowing. An oral cavity and throat examination using penlight illumination revealed throat inflammation and swelling. His cervical echocardiography showed no abnormal findings, but auscultation of his bilateral chest recorded discontinuous sound. He was diagnosed with acute pharyngitis and prescribed 500 mg/day of levofloxacin and 600 mg/day of acetaminophen. On Day 20, his sore throat worsened. Polymerase chain reaction tests using a throat swab were negative for influenza A, influenza B, COVID‐19, and tuberculosis. We decided that the patient had more than acute pharyngitis. Our hospital is a psychiatric hospital primarily responsible for alcoholism treatment and does not have an otolaryngology department. Therefore, we consulted a neighboring ear and nose clinic (Clinic “A”). The doctor of Clinic “A” identified a mass in the patient's pharynx via endoscopy and decided that the patient was at risk of airway obstruction and needed close examination. Therefore, he was referred to the otolaryngology department of General Hospital “B” on Day 29 of his hospitalization.

**Figure 1:**
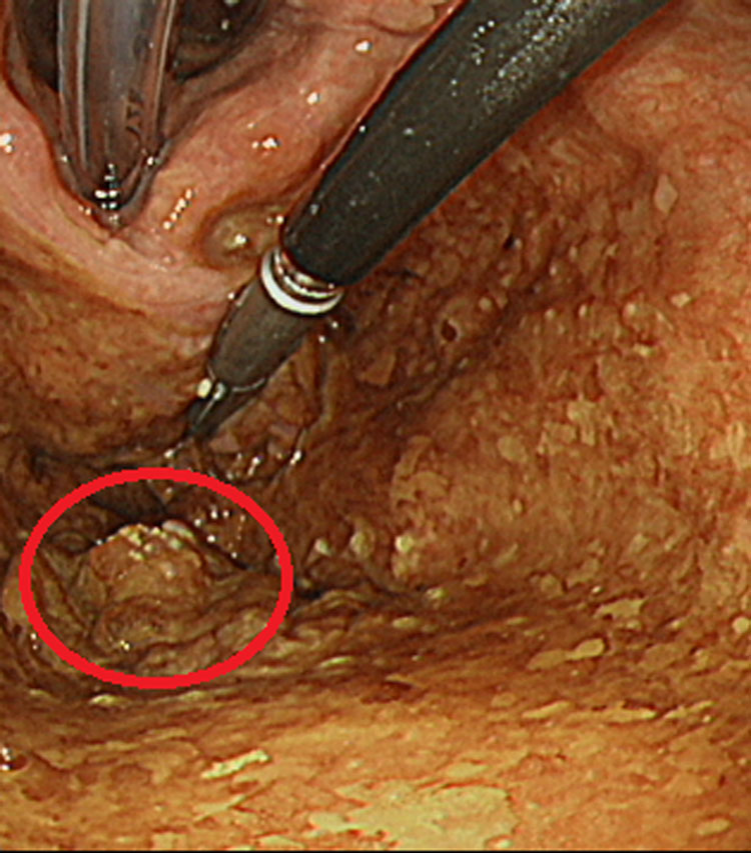
The endoscopy shows a borderline indistinct elevation in the patient's hypopharynx (red oval). This image was taken at the time the tumor was resected.

**Figure 2:**
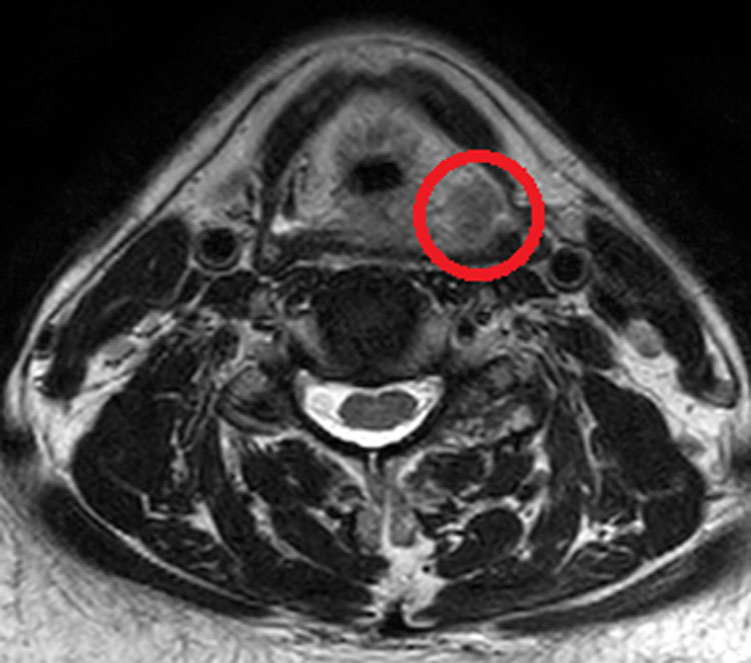
The patient's head MRI showed a nodule (red oval) behind the trachea.

**Figure 3:**
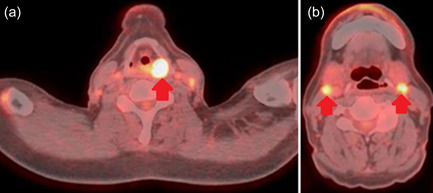
The patient's 18F‐fluorodeoxyglucose positron emission tomography (FDG)/computed tomography showed high FDG accumulation (a) behind the trachea (red arrow) and (b) in the surrounding lymph nodes (red arrows).

Imaging examination at Hospital “B” exposed important abnormalities. His endoscopy results showed a mass in his pharynx (Figure 1, red oval). His neck magnetic resonance imaging results showed a 2 × 2 × 2‐cm nodule above the left glottis (red oval in Figure 2), and his 18F‐fluorodeoxyglucose positron emission tomography/computed tomography (18F‐FDG PET/CT) results showed FDG accumulation with a standardized uptake value of 18.4 at that site (Figure 3a, red arrow). These findings were suggestive of malignancy. In the regional lymph nodes, we observed FDG accumulation in the bilateral superior internal deep cervical and submandibular lymph nodes (Figure 3b, red arrow). This finding was suggestive of lymph node metastasis.

On the basis of the imaging and pathology results detailed above, the patient was diagnosed with Stage 4 A (T2 N2c M0) hypopharyngeal cancer. The psychiatrists and otolaryngologists of Hospital “B” discussed not only the patient's physical condition but also his mental and psychosocial state. The otolaryngologists were particularly concerned about the extent of the patient's alcoholism and decision‐making capacity. They were also concerned that delirium tremens could lead to accidents during the perioperative period. General Hospital “B” has internal medicine, surgery, and otorhinolaryngology departments, but does not have a psychiatric department. We explained that the patient had previously been sober for extended periods of time, and his prospects of recovery from alcohol dependence were good. We also explained that the patient was no longer in a critical period of delirium tremens. In addition, the patient's Hasegawa Dementia Scale‐Revised (HDS‐R) and Mini Mental State Examination (MMSE) scores were determined; his HDS‐R score was 27/30, and his MMSE score was 28/30. On the basis of these test results and the patient's daily life, we concluded that the patient did not have dementia. Furthermore, the patient had no symptoms suggestive of mood disorders and psychosis. On the basis of these results, the patient was considered to have decision‐making capacity. With regard to financial issues, we assisted the patient in applying for state support to help him with follow‐up after his cancer treatment. We worked with the patient on the decision‐making process. The patient was given careful information by the otolaryngologist regarding the recommended surgical treatment and postoperative radiotherapy. However, the patient was very anxious and could not immediately make a decision about his treatment plan. Therefore, the psychiatrist, caseworkers, and nurses attended to his concerns and helped to calm him. His worst fear was that the highly invasive surgery would damage his speech functions, reduce his ability to communicate, and make him feel alone. His second worst fear was the pain associated with radiotherapy. However, he understood that the inconvenience of living after lymphadenectomy would be alleviated by rehabilitation.

He was therefore reassured and decided to undergo surgery. Finally, we decided on a treatment strategy of endoscopic laryngopharyngeal surgery (ELPS) combined with lymphadenectomy. To reduce invasiveness, the surgery was divided into two sessions, ELPS and lymphadenectomy.

On Day 86, the patient underwent ELPS. Postoperatively, he still had a sore throat and decreased appetite; therefore, we increased the dose of acetaminophen to 1800 mg/day. In addition, we administered 200 mg of tramadol. Because hard food made his sore throat worse, we offered soft food and nutritional drinks. He gradually regained his vitality, and his weight recovered to 76 kg. We decided to introduce a home nursing service and prescribe day care to support his discharge and improve his postoperative quality of life. Lymphadenectomy was carried out on Day 114. His postoperative recovery was good. He is now in our hospital undergoing rehabilitation and preparing for discharge.

## DISCUSSION AND CONCLUSION

In the case examined here, the time from sore throat to examination and diagnosis of hypopharyngeal cancer was 13 days. This very rapid response likely contributed to the good outcome. Although their usefulness is debatable, the London urgent referral guidelines for patients with suspected head and neck cancer recommend that the waiting period between examination and diagnosis should be no longer than 2 weeks.^5^

During psychotherapy, the patient's alcohol dependence was treated with counseling by the attending psychiatrist and with group therapy, such as Alcoholics Anonymous meetings. Group therapy, in particular, was somewhat effective; although it did not keep the patient sober, it did prevent social isolation and inexhaustible drinking. However, the COVID‐19 pandemic often prevented group therapy from taking place. This resulted in periods when patients were not able to receive adequate psychotherapy, which is considered to be an important issue in alcoholism treatment.

Benzodiazepines, such as diazepam, are often used to treat delirium tremens in patients with alcoholism.^6^ However, in this case, the patient's consciousness was only mildly affected. He also did not show any hallucinations or agitation. Therefore, we did not prescribe benzodiazepines and focused on physical treatment, such as intravenous infusions containing B vitamins.

The role played by psychiatrists in this case was twofold. The first was to assist with otolaryngological treatment by treating delirium tremens and assessing decision‐making capacity. The patient was a single man and had no family or friends to support him with regard to treatment decisions; therefore, we took on that role. In this case, the patient's decision‐making capacity was assessed in open and closed interviews.^7^ Specifically, the open question was “What explanation did you receive about your disease?” (in Japanese). Closed questions were used to complement the open questions, for example, more detailed information on the cancer stage and the risks and benefits of treatment options.

His answers to these questions matched the explanations given by the otolaryngologist to our psychiatric staff. Therefore, we judged the patient to have decision‐making capacity. The second role was to support the patient and empathize with his experience of anxiety and depression. The difficulties in psychotherapy for alcoholics have complex causes, including the patient's decision‐making capacity, self‐care capacity, and the non‐establishment of a therapeutic relationship. In particular, the patient had to overcome anxiety about the uncertain schedule of the operation because the COVID‐19 pandemic had restricted surgical treatment at the general hospital. During this period, he sometimes showed symptoms of anxiety and depression. We endeavored to listen to him. This case report suggests that knowledge and practice of palliative care are needed by all psychiatrists, not only psychiatrists specializing in psycho‐oncology who are involved in liaison consultation at general hospitals. It is considered necessary for all psychiatrists to be interested not only in psychotherapy but also in the physical treatment options of the patient. It is also necessary to discuss these options with the oncologist without reservation.

These efforts will surely lead to beneficial results in the treatment of patients' physical and mental health, in our opinion.

## AUTHOR CONTRIBUTIONS

Ishida T was the patient's primary care physician and contributed to the literature review and manuscript preparation. Murayama T and Kobayashi S reviewed the literature and contributed to the preparation of the manuscript. All authors gave their approval for the final version to be submitted.

## CONFLICT OF INTEREST

The authors declare no conflict of interest.

## ETHICS APPROVAL STATEMENT

This study was conducted according to the principles of the Declaration of Helsinki.

## PATIENT CONSENT STATEMENT

Informed written consent was obtained from the patient for publication of this report and any accompanying images.

## CLINICAL TRIAL REGISTRATION

Not applicable as this is a case report.

## Data Availability

Not applicable as this is a case report.
